# Predicting the risk of distant metastasis in patients with locally advanced rectal cancer using model based on pre-treatment T2WI-based radiomic features plus postoperative pathological stage

**DOI:** 10.3389/fonc.2023.1109588

**Published:** 2023-09-07

**Authors:** Chen Wang, Jingjing Chen, Nanxin Zheng, Kuo Zheng, Lu Zhou, Qianwen Zhang, Wei Zhang

**Affiliations:** ^1^ Department of Colorectal Surgery, Changhai Hospital, Naval Medical University, Shanghai, China; ^2^ Hereditary Colorectal Cancer Center and Genetic Block Center of Familial Cancer, Changhai Hospital, Shanghai, China; ^3^ Graduate School of Naval Medical University, Shanghai, China; ^4^ Department of Radiology, Changhai Hospital, Naval Medical University, Shanghai, China

**Keywords:** locally advanced rectal cancer, radiomics feature, nomogram, distant metastasis free survival, neoadjuvant chemoradiotherapy, radiomic feature

## Abstract

**Objective:**

To assess the prognostic value of a model based on pre-treatment T2WI-based radiomic features and postoperative pathological staging in patients with locally advanced rectal cancer who have undergone neoadjuvant chemoradiotherapy.

**Methods:**

Radiomic features were derived from T2WI, and a radiomic signature (RS) was established and validated for the prediction of distant metastases (DM). Subsequently, we designed and validated a nomogram model that combined the radiomic signature and postoperative pathological staging for enhanced DM prediction. Performance measures such as the concordance index (C-index) and area under the curve (AUC) were computed to assess the predictive accuracy of the models.

**Results:**

A total of 260 patients participated in this study, of whom 197 (75.8%) were male, and the mean age was 57.2 years with a standard deviation of 11.2 years. 15 radiomic features were selected to define the radiomic signature. Patients with a high-risk radiomic signature demonstrated significantly shorter distant metastasis-free survival (DMFS) in both the development and validation cohorts. A nomogram, incorporating the radiomic signature, pathological T stage, and N stage, achieved an area under the curve (AUC) value of 0.72 (95% CI, 0.60-0.83) in the development cohort and 0.83 (95% CI, 0.73-0.92) in the validation cohort.

**Conclusion:**

A radiomic signature derived from T2WI-based radiomic features can effectively distinguish patients with varying risks of DM. Furthermore, a nomogram integrating the radiomic signature and postoperative pathological stage proves to be a robust predictor of DMFS.

## Introduction

Colorectal cancer ranks as the third most common cancer globally and stands as the second leading cause of cancer-related mortality worldwide (1). Rectal cancers constitute approximately 30% of all colorectal malignancies (1–3). For patients diagnosed with locally advanced rectal cancer (LARC), the current standard treatment protocol involves neoadjuvant chemoradiotherapy (nCRT) followed by total mesorectal excision (TME) (4, 5). Despite the significant reduction in local recurrence (LR) rates, there is not necessarily a corresponding improvement in overall survival. Distant metastasis (DM) remains the primary cause of treatment failure in LARC (4, 6). Consequently, the accurate prediction of a patient’s risk of DM becomes crucial in tailoring appropriate treatments and enhancing oncological outcomes for LARC.

Similar to other malignancies, rectal cancer exhibits a tendency to metastasize to distant organs from the onset of primary lesion formation (7). This metastatic capability is closely tied to the features of the primary lesion (8). However, current practices primarily rely on histopathological examination of surgical specimens to assess the risk of distant metastasis. These pathological factors, mostly derived after nCRT, fail to evaluate the intrinsic biological heterogeneity of LARC prior to treatment, which theoretically has a close association with distant metastasis. Consequently, they fall short in providing comprehensive prognostic information about distant metastasis. Therefore, the integration of postoperative pathological factors with pre-treatment noninvasive prognostic biomarkers to assess tumor heterogeneity could be a valuable approach for facilitating personalized medicine.

Nowadays, magnetic resonance imaging (MRI) is routinely utilized for staging prior to nCRT in the treatment of LARC. This allows for an assessment of the biological heterogeneity of LARC before nCRT (9, 10). MRI-based radiomics is a non-invasive, high-throughput post-processing technique that extracts a vast number of quantitative features from standard medical images (11). Through the measurement of gray level distributions and relationships within a lesion, radiomic texture features can expose non-visual information related to tumor heterogeneity and its microenvironment. This results in a detailed and comprehensive characterization of the tumor phenotype (12).

The primary objective of this study is to investigate the relationship between pretreatment T2WI-based radiomic features and postoperative pathological staging in correlation with the risk of DM in patients with LARC. Furthermore, we aim to develop a model for personalized prediction of risk of DM, which can serve as a guide for precision medicine.

## Methods

### Participant inclusion

Patients with rectal adenocarcinoma treated in the department of colorectal surgery, Changhai Hospital between January 2010 and December 2018 were reviewed. The inclusion criteria were as followed: (1) LARC was determined at pre-nCRT MRI (stage pretreatment T2 weighted MR images as cT3/T4, and/or N-category positive); (2) patients received pretreatment multiparameter MRI, including high-resolution T2WI MR imaging; (3) patients were treated by long-course nCRT followed by radical TME surgical resection; (4) the LARC was the first and only malignant tumor; (5) patients received 5-fluorouracil-based adjuvant chemotherapy for 4~8 times after surgery. The exclusion criteria were as follows: (1) the quality of MR image was poor; (2) an interval longer than 16 weeks between the completion of neoadjuvant radiotherapy and surgery; (3) the followed-up time was less than 3 months.

Informed consent was obtained from each patient before nCRT and surgery. The clinicopathologic and follow-up data of all patients were collected from the prospectively maintained colorectal cancer database of Changhai Hospital, Shanghai, China. And this study was approved by the Institutional Review Board of Changhai Hospital, Secondary Military Medical University, Shanghai, China.

### Neoadjuvant treatment and surgery

All patients underwent three-dimensional conformal radiation therapy (gross tumor volume, 45-50.4 Gy; clinical target volume, 1.8-2.0 Gy; a total of 25-28 fractions). Concomitantly, capecitabine (800 mg/m2 orally twice daily) was administered with radiation therapy. After radiation therapy, patients received 5-fluorouracil-based consolidated chemotherapy for 0-3 times. TME surgery was performed with 4 to 16 weeks after the completion of radiation therapy. Afterward, patients received 5-fluorouracil-based adjuvant chemotherapy for 4-8 times.

Surgically resected specimens were evaluated by two specialist colorectal cancer pathologist, according to the Seventh American Joint Committee on Cancer (AJCC) TNM system, and the discrepancies were settled by a senior pathologist. The data about tumor staging, lymph node involvement, lymphovascular invasion (LVI), and perineural invasion (PNI) were retrospectively collected. The nCRT response was evaluated according to the 7th AJCC/NCCN tumor regression grade (TRG) scale.

### Clinical endpoints and follow-up

Patients were followed up regularly after surgery by telephone contacts or interviews in outpatient clinic, with 3-month intervals for the first 2 years, then 6-month intervals for the 3rd to 5th years, and annually thereafter. The endpoint of this study was distant metastasis free survival (DMFS), which was measured from the date of surgery to the first distant metastasis, death from any cause, or the last visit in follow up (censored), and the models were built based on the DMFS. The distant metastasis was confirmed by clinical examination, imaging methods such as chest computerized tomography (CT), and abdominopelvic CT or MRI, or biopsy proven.

### MRI protocol and radiomics analysis

MRI was performed with the use of a 3.0-T MRI scanner (Phillips Healthcare). Detailed information on MRI protocol was presented in **Supplementary Table 1**.

**Figure 1:**
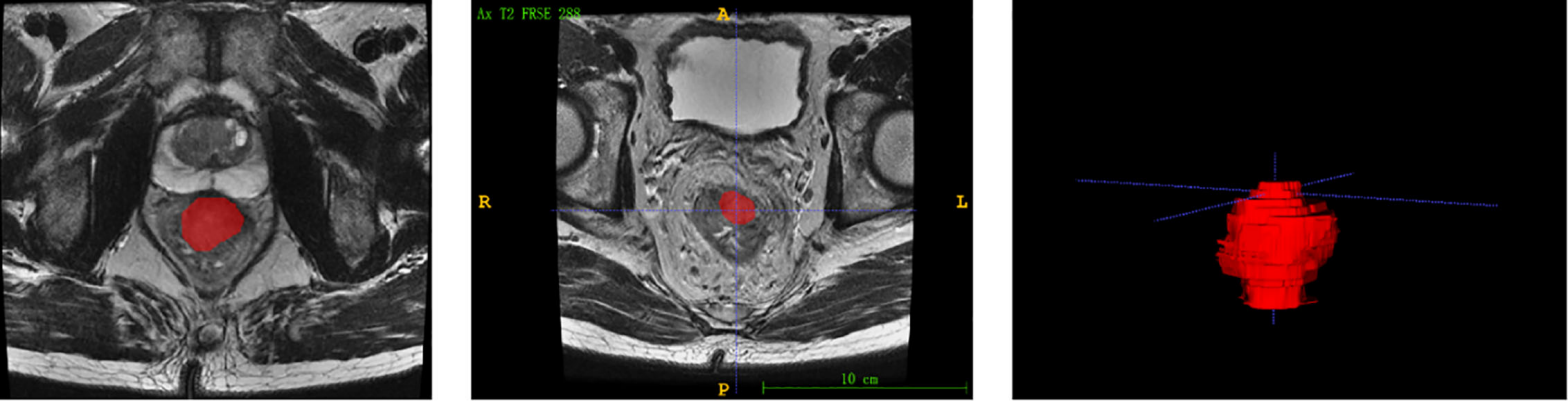
The segmentation and delineation of regions of interest (ROIs).

Each patients’ MRI data were collated for tumor masking and feature extraction. The regions of interest (ROIs) were delineated manually using the itkSNAP software (www.itksnap.org). Radiomic feature extraction was performed by a panel of radiologists (Nanxin Zheng, Jingjing Chen and Chen Wang) using 3D Slicer version 4.10 (www.slicer.org), a free and open-source software, to semiautomatically segment the entire area after treatment within the rectal wall, excluding equivocal normal rectal wall and mucosal edema on the high-spatial resolution axial T2-weighted images, as shown in **Figure 1**.

### Radiomic feature extraction and radiomic model building

The kinds of features extracted from MR image included shape, the first-order statistic, texture features (grey-level size zone matrix; grey-level co-occurrence matrix; grey-level dependence matrix; grey-level run-length matrix), and 2107 features were obtained. Only the radiomic features with good interobserver reproducibility (intraclass correlation coefficient [ICC] >0.8) were included in subsequent analyses, and 1158 features were included in the next analyses.

Univariate Cox analysis was initially used to detect the associations between each feature and the patients’ DMFS. and the top 20% of the features with P< 0.15 were used for further analysis. Among those features, the Pearson correlation coefficients (r) for each feature pair were than calculated. Feature pairs with |r|>0.5 were selected, and then in each of these pairs, the feature with larger mean absolute correlation was removed. Finally, the least absolute shrinkage and selection operator (LASSO) algorithm with Cox analysis was conducted to choose the optimized subset of features to construct the final model in the development cohort, and radiomic signatures was computed.

### Statistical analyses

All statistical analyses were performed by using R software (version 4.0.2, http://www.R-project.org). The differences in patient characteristics data between the training and validation cohorts were assessed by using Student t test, Man-Whitney U test, Chi-Squared tests, or Fisher exact test, where appropriate. Kaplan-Meier survival curves and the log-rank test were used to compare differences in the survival. A calibration curve was employed to calibrate the nomogram. Receiver operating characteristic (ROC) curve analysis was performed to evaluate the model’s prediction power. A two-sided P value of <0.05 was considered statistically significant.

## Results

### Patients’ baseline characteristics

A total of 260 patients were enrolled in this study, including 197 (75.8%) men, 63 (24.2%) women. The mean age was 57.2 (11.2) years. The median follow-up period was 41.1 (IQR 27.5-54.8) months. The patients were randomly divided into a development cohort (n=156) and a validation cohort (n=104) at a ratio of 3:2. There was no significant difference between two sets in baseline demographic clinicopathological characteristics, as shown in **Table 1**.

**Table 1:** The baseline characteristics of patients in this study.

Characteristic	Development cohort (n=156)	Validation cohort (n=104)	*P* value
Age, yrs, mean (SD)	56.5(11.4)	58.2(10.9)	0.247
Sex, female	35(22.4)	28(26.9)	0.497
BMI, kg/m^2^, mean (SD)	23.3(3.3)	23.2(2.9)	0.894
Family history of maligancy, yes	13(8.3)	3(2.9)	0.376
Concomitant disease, yes	49(31.4)	30(28.8)	0.762
CEA, up	37(23.7)	24(23.1)	0.999
CA199, up	12(7.7)	7(6.7)	0.961
Gross appearance			0.999
Ulcerative	119(76.3)	80(76.9)	
Polypoid	37(23.7)	24(23.1)	
Tumor height			0.389
≥5cm	55(35.3)	43(41.3)	
<5cm	101(64.7)	61(58.7)	
pTRG			0.529
0	27(17.3)	15(14.4)	
1	37(23.7)	30(28.8)	
2	43(27.6)	33(31.7)	
3	49(31.4)	26(25.0)	
Differentiation			0.826
Disappear	27(17.3)	15(14.4)	
Well/moderately	29(18.6)	20(19.2)	
Poor	100(64.1)	69(66.3)	
Pathological T stage			0.155
0	27(17.3)	15(14.4)	
1/2	45(28.8)	42(40.4)	
3/4	84(53.8)	47(45.2)	
Pathological N stage			0.390
0	115(73.7)	79(76.0)	
1	28(17.9)	13(12.5)	
2	13(8.3)	12(11.5)	
LVI^1^, yes	6(3.8)	3(2.9)	0.945
PNI^2^, yes	17(10.9)	12(11.5)	0.999
R0 resection, no	6(3.8)	1(1.0)	0.310
Tumor budding, yes	12(7.7)	7(6.7)	0.961
TD^3^, yes	18(11.5)	16(15.4)	0.476

LVI, lymphovascular invasion.

PNI, perineural invasion.

TD, tumor deposit.

### Radiomic signature construction and validation

**Figure 2:**
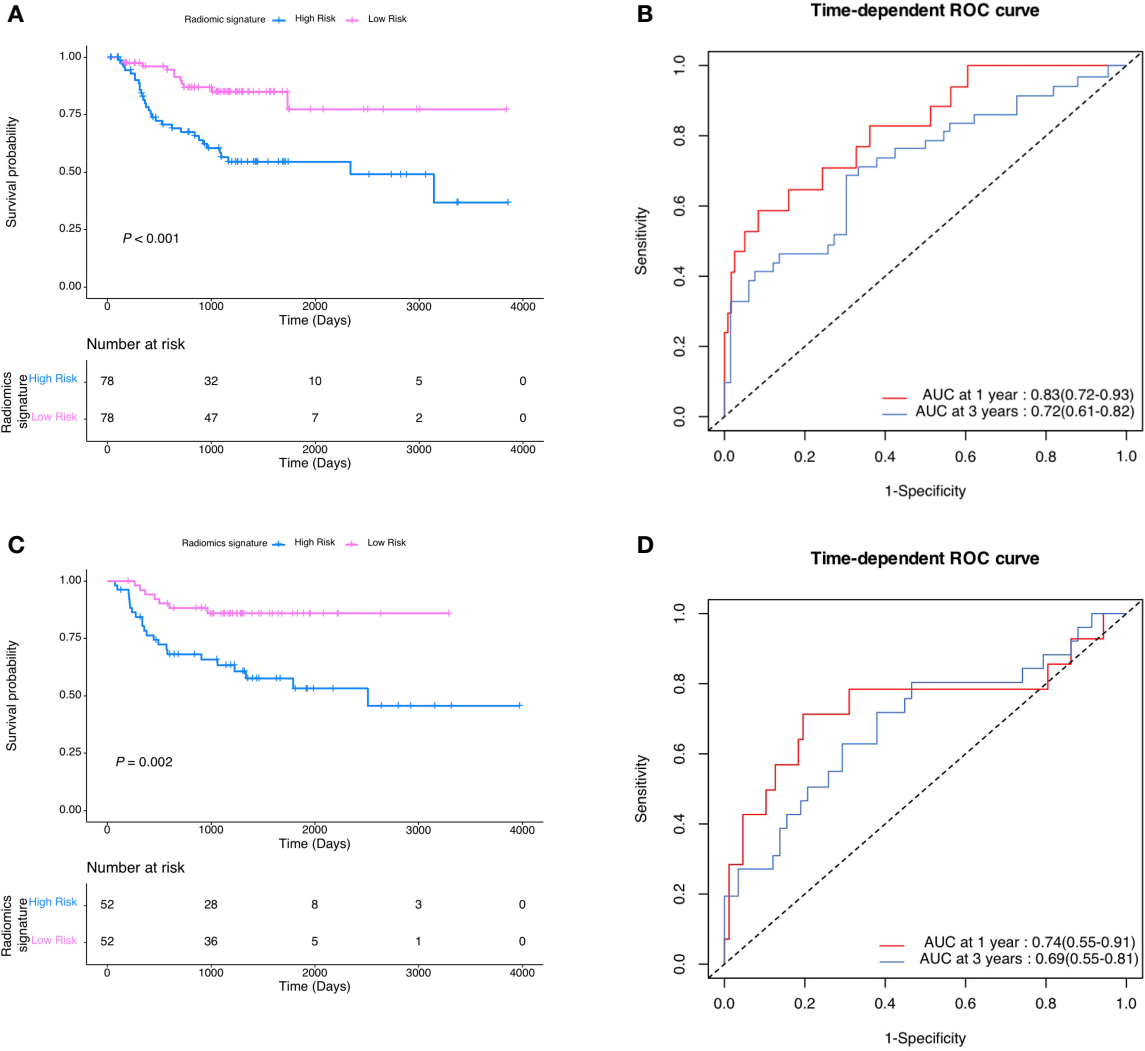
**(A)** DMFS according to radiomic signature in the development cohort. **(B)** Radiomic signature estimated DMFS in development cohort. **(C)** DMFS according to radiomic signature in the validation cohort. **(D)** Radiomic signature estimated DMFS in validation cohort.

After coarse-to-fine feature selection strategy as stated in methods, 15 radiomics features were selected and then incorporated into a LASSO-Cox regression model to define the radiomic signature, as shown in **Table 2**. Finally, As showed in **Figure 2**, there was a statistically significant difference in DMFS between patients with high risk radiomic signature and those with low risk radiomic signature (P<0.001). The radiomic signature had area under the curve (AUC) values of 0.83 (95%CI 0.72 to 0.93) for 1-year DMFS and 0.72 (95%CI 0.61 to 0.82) for 3-year DMFS. In the validation cohort, there was also a statistically significant difference in DMFS between patients with high risk radiomic signature and those with low risk radiomic signature (P=0.002). The radiomic signature had area under the curve (AUC) values of 0.74 (95%CI 0.55 to 0.91) for 1-year DMFS and 0.69 (95%CI 0.55 to 0.81) for 3-year DMFS.

**Table 2:** The selected features and associated coefficients.

Features	Coefficients
original_shape_MajorAxisLength	0.68198
log_sigma_2_0_mm_3D_glrlm_RunPercentage	1.69065
log_sigma_3_0_mm_3D_glszm_GrayLevelNonUniformity	2.36652
log_sigma_4_0_mm_3D_gldm_LargeDependenceHighGrayLevelEmphasis	2.68304
log_sigma_5_0_mm_3D_firstorder_90Percentile	0.46904
wavelet_LLH_glcm_MCC	1.00224
wavelet_LHL_glcm_MCC	1.13433
wavelet_LHH_firstorder_Kurtosis	2.51254
wavelet_LHH_glszm_GrayLevelNonUniformityNormalized	1.67246
wavelet_LHH_glszm_LargeAreaLowGrayLevelEmphasis	3.19551
wavelet_LHH_glszm_SizeZoneNonUniformityNormalized	0.87635
wavelet_HLL_gldm_SmallDependenceHighGrayLevelEmphasis	3.33897
wavelet_HLH_firstorder_Skewness	0.52339
wavelet_HHL_glszm_ZoneVariance	2.04540
wavelet_LLL_gldm_LargeDependenceLowGrayLevelEmphasis	8.05213

### Prognostic nomogram for DMFS

**Figure 3:**
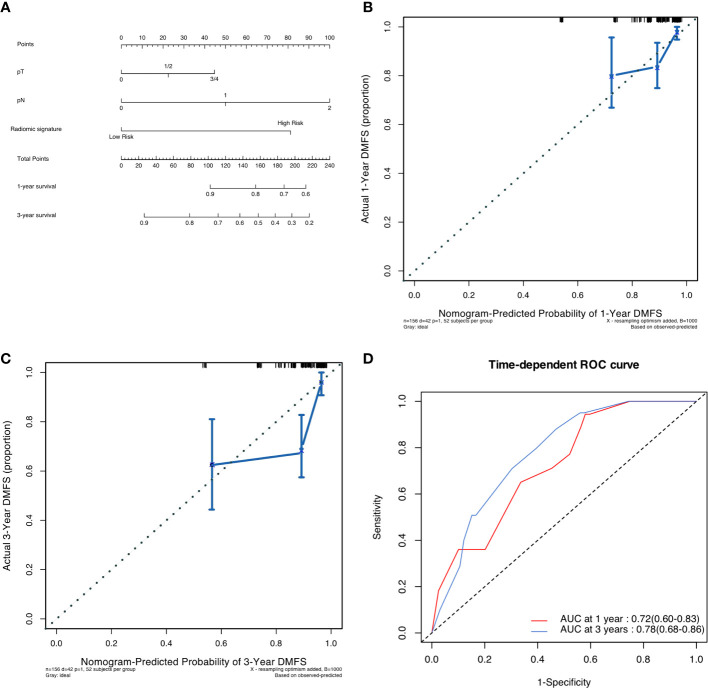
**(A)** Pathological-radiomic nomogram. **(B)** Calibration curve for DMFS at 1 years in the development cohort. **(C)** Calibration curve for DMFS at 3 years in the development cohort. **(D)** Time-dependent ROC curve of nomogram in development cohort.

As shown in **Figure 3**, a pathological-radiomic nomogram combining conventional pathological T stage, pathological N stage and radiomic signature in the development cohort was built and the C-index was 0.747 (95%CI 0.711 to 0.783), higher than that of a nomogram based on conventional pathological T stage and N stage (0.696, 95%CI 0.655 to 0.737). The calibration plot for the probability of survival at 3 or 5-year after surgery showed an optimal agreement between the prediction by nomogram and actual observation. The pathological-radiomic nomogram had AUC values of 0.72 (95%CI 0.60 to 0.83) for 1-year DMFS and 0.78 (95%CI 0.68 to 0.86) for 3-year DMFS.

### Validation of predictive accuracy of the nomogram for DMFS

**Figure 4:**
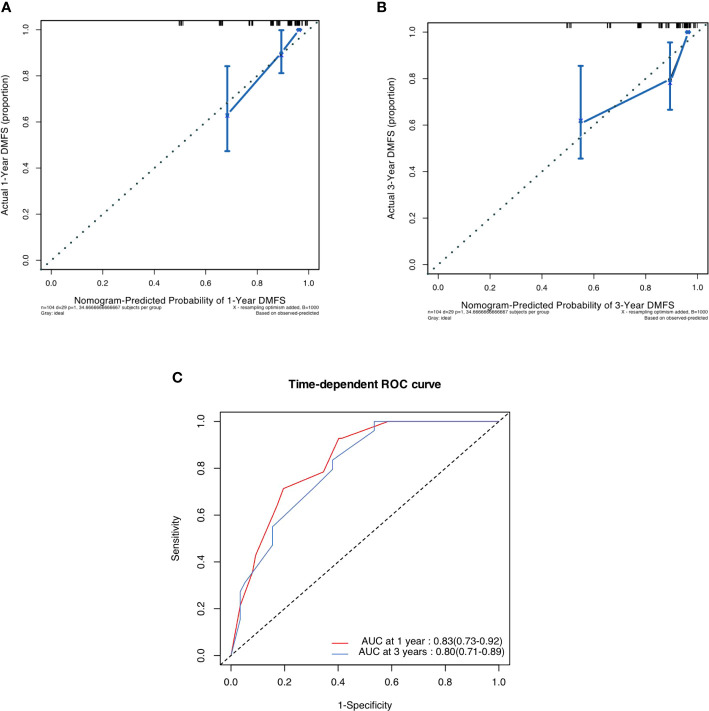
**(A)** Calibration curve for DMFS at 1 years in the validation cohort. **(B)** Calibration curve for DMFS at 3 years in the validation cohort. **(C)** Time-dependent ROC curve of nomogram i.

In the validation cohort, the median follow-up time was 43.3 (IQR 34.6 to 60.7) months. The postoperative 1- and 3- DMFS rates were 86.3% (95%CI 79.8% to 93.2) and 74.7% (66.5% to 83.8%), respectively. In the validation cohort, the C-index of pathological radiomic nomogram for predicting DMFS was 0.788 (95%CI 0.751 to 0.825), higher than that of a nomogram based on conventional pathological T stage and N stage (0.761, 95%CI 0.720 to 0.802). As shown in **Figure 4**, a calibration curve showed good agreement between prediction and observation in the probability of 1-year and 3-year DMFS. The pathological-radiomic nomogram had AUC values of 0.83 (95%CI 0.73 to 0.92) for 1-year DMFS and 0.80 (95%CI 0.71 to 0.89) for 3-year DMFS.

## Discussion

In this study, we constructed a prognostic index, termed a “radiomic signature,” derived from T2WI radiomic features. This radiomic signature was then integrated with the conventional pathological staging system to create a pathological-radiomic nomogram for the prediction of DMFS in rectal cancer patients undergoing nCRT and surgery. The radiomic signature and the pathological-radiomic nomogram were validated in the validation cohort, demonstrating no significant statistical differences when compared to the development cohort. The results indicate that the radiomic signature offers superior prognostic discrimination. Moreover, the pathological-radiomic nomogram demonstrated superior performance over the conventional pathological staging system in predicting DMFS in both development and validation cohorts.

At present, the prognostic prediction of patients received nCRT and subsequent surgery is unsatisfactory, which partly resulted from the impact of nCRT (13). Some clinicians insisted that all locally advanced rectal cancer patients receiving nCRT were supposed to receive adjuvant chemotherapy after surgery regardless of the response towards to nCRT, due to the advanced stage of those patients at the diagnosis (14–16). Jung et al. reported a study including 551 patients concluded that adjuvant chemotherapy was significantly associated with increased DFS among patients who had undergone nCRT and surgery for LARC (17). Dossa et al. reported that the adjuvant chemotherapy even improved the overall survival of patients with rectal cancer with pCR, particularly those with pretreatment node-positive disease (18). This study implied the pretreatment clinicopathological features also were of assistance to guide of adjuvant therapy. However, a pooled analysis indicated that pCR patients would not benefit form adjuvant chemotherapy (19). The conflicting evidence of whether patients after nCRT and surgery would benefit from adjuvant therapy would result from the underestimation of detection towards tumor biology before nCRT.

In the era of precision medicine, predictive models anchored in biomarker data are becoming increasingly vital in pioneering personalized treatment plans and sophisticated therapies, including anti-angiogenic and immunotherapy (20, 21). However, these models frequently depend on biopsy samples that inherently offer only a limited spatial portrayal of lesions (22). This restricted sampling can potentially instigate a level of bias, occasionally resulting in false-negative diagnoses in the realm of tumor detection and characterization (23).

With the advance of theory and technique in radiology, radiomics is estimated as an effective, noninvasive method to detect the detailed and comprehensive characterization of the tumor (24). The prognostic value radiomics features has been proved in many other kinds of tumors including hepatocellular cancer, breast cancer and pancreatic cancer (24–26). In our study, T2WI-based radiomic features served as an ideal technique extracting large amounts of quantitative features from images of treatment-naive rectal cancer. After careful selection of features, radiomic signature was built to better predict the DMFS of rectal cancer patients. The pathological-radiomic nomogram also performed better than conventional pathological stage system.

The development of treatment-naive radiomic signature and pathological-radiomic nomogram has allowed for the identification of low-risk and high-risk rectal cancer patients receiving nCRT and surgery (27). To our knowledge, there is currently little use of treatment-naive radiomics combining with pathological features to predict oncologic outcomes of patients receiving nCRT and surgery. As MRI is routinely recommended before the nCRT, our study provides a new method to risk stratification for rectal patients. In addition, as MRI is routinely recommended before the nCRT, images are already widely available, the radiomic signature and pathological-radiomic nomogram could be updated conveniently and improve rectal cancer management more rapidly than other markers like molecular.

The tumor microenvironment (TME) encompasses the non-malignant cellular components within and surrounding the tumor, including but not limited to immune cells, fibroblasts, vascular structures, and the extracellular matrix. These elements collectively exert significant influence on tumor behavior and its response to therapeutic interventions. It is well-documented across various cancer types that the TME substantially impacts the propensity for distant metastases (28–30). Despite these findings, the association between the radiomic signature and the TME is yet to be fully elucidated. In our forthcoming research, we plan to investigate this relationship further, aiming to deepen our comprehension of tumor biology and potentially inform the development of innovative therapeutic strategies.

This study has several limitations. Firstly, it is a single-center study, thus the external validity of the pathological-radiomic nomogram remains to be established. Future studies should incorporate data from multiple centers or leverage publicly available datasets, such as The Cancer Imaging Archive (TCIA), for further validation of our proposed nomogram. Secondly, a substantial number of radiomic features demonstrated low inter-observer agreement and were consequently excluded from our study. To mitigate this issue, we plan to enhance our workflow to minimize interobserver variability in future research. Lastly, the radiomic features for this study were derived solely from T2WI, which may not capture the full spectrum of lesion information. Subsequent studies should consider performing radiomic analysis using multiple imaging sequences to provide a more comprehensive characterization of the lesions.

## Data availability statement

The data analyzed in this study is subject to the following licenses/restrictions: The analysis data supporting the findings of this study are available from the corresponding author Wei Zhang on reasonable request. Requests to access these datasets should be directed to Wei Zhang, weizhang2000cn@163.com.

## Ethics statement

The studies involving human participants were reviewed and approved by the Ethics Committee of Changhai Hospital. The patients/participants provided their written informed consent to participate in this study.

## Author contributions

NZ conceived the research question, study design, supervised on methodology and technical details, and reviewed the manuscript. JC and CW contributed to the research question, study design, conducted the study, and drafted original manuscript. KZ and LZ contributed to data extraction, validation, analysis and visualization on the manuscript. WZ and QZ provided critical consultation and interpretation from clinical perspective and contributed to manuscript writing. All authors contributed to the article and approved the submitted version.
