# Supplementary material for: Predicting the risk of distant metastasis in patients with locally advanced rectal cancer using model based on pre-treatment T2WI-based radiomic features plus postoperative pathological stage
Source: Front Oncol. 2023 Sep 7;13:1109588. doi: 10.3389/fonc.2023.1109588 (PMC10517628; doi:10.3389/fonc.2023.1109588)
Supplement: Supplementary file 1 [file Table_1.docx]

**Supplemental Table 1.** The main sequence protocol and parameters of MRI

|  | **Parameter** |
| --- | --- |
| **Magnetic field strength** | 3.0T |
| **Oblique axial T2WI** |  |
| Echo train length | 32 |
| Field of view (mm) | 200×200 |
| Section thickness (mm) | 4 |
| Matrix | 352×352 |
| TR/TE^1^ (ms) | 6538/116 |
| Bandwidth (kHz) | 62.5 |
| Flip angle (°) | 110 |
| **Axial DWI** |  |
| Field of view (mm) | 320×256 |
| Section thickness (mm) | 6 |
| Matrix | 128×128 |
| TR/TE (ms) | 2840/Minimum |
| b values (s/mm^2^) | 0, 1000 |

1. TR/TE, Repetition time/echo time.
